
JOURNAL INFORMATION
==============================
NLM Title Abbreviation: Schizophr Bull
Journal ID: Schizophr Bull
NLM Journal ID: 0236760
Journal ID: schbul

Schizophrenia Bulletin

ISSN: 0586-7614
EISSN: 1745-1701
Publisher: Oxford University Press

ARTICLE INFORMATION
==============================
PMCID: PMC5888590
DOI: 10.1093/schbul/sby018.849
Article ID: sby018.849
Article version: 1
Subjects: Abstracts, Poster Session III

S62. Submission Withdrawn

Print publication date: 2018 Apr
Electronic publication date: 2018 Apr 1
Volume: 44
Issue: Suppl 1
Issue title: Abstracts for the Sixth Biennial SIRS Conference
First page: S438
Last page: S438
Copyright: © Maryland Psychiatric Research Center 2018.
Copyright year: 2018
License: This is an Open Access article distributed under the terms of the Creative Commons Attribution License (http://creativecommons.org/licenses/by/4.0/), which permits unrestricted reuse, distribution, and reproduction in any medium, provided the original work is properly cited.
License URL: https://creativecommons.org/licenses/by/4.0/

Page count: 1

==============================
**Methods:** The SFRT-2 was translated and retro-translated into Spanish. After that, one hundred and one patients with schizophrenia and 100 HC were assessed in order to obtain psychometric properties of the test. First, reliability of the SFRT-2 was studied with Cronbach's alpha coefficients for actions hits, actions false positives, goals hits and goals false positives separately, in both patients and HC. Second, in patients' group, concurrent validity was calculated using Spearman's correlations in order to assess the relationship between SFRT hits and false positives scores and other SC measures such as ToM, EP, AS and global SC. Third, divergent validity was assessed in patients' group by means of Spearman's correlations in order to study the relationship between SFRT-2 and a neurocognition composite score. Finally, discriminant validity of SFRT-2 actions and objectives hits and false positives was obtained comparing schizophrenia and HC groups by means of Receiver Operating Characteristic (ROC) curve analysis. Percentiles for the SFRT-2 scores were also calculated and shown in order to facilitate clinical assessment of SP.
**Results:** Regarding reliability of the test, internal consistency indexes of the SFRT-2 hits and false positives ranged from $\alpha = .66$ to $\alpha = .90$ in both groups, with higher indexes corresponding to patients' group. Concerning convergent and divergent validity, SFRT-2 significantly correlated with other measures of SC, especially with ToM (SFRT-2 hits: $r = .46$, $p < .01$), and also, but to a lesser extent, with neurocognition composite score (SFRT-2 hits: $r = .33$, $p < .01$). Receiver Operating Characteristic (ROC) curve analysis showed that SFRT-2 hits and false positives discriminate well between patients with schizophrenia and HC, being false positives the indexes which best discriminated between both groups (actions false positives: AUC = .74, $p < .001$; objectives false positives: AUC = .78, $p < .001$).
**Discussion:** Spanish adaptation and validation of the SFRT-2 showed good psychometric properties in both patients with schizophrenia and HC. In addition, reliability of the instrument seemed to be especially high among patients with schizophrenia. To our knowledge, this is the first adaptation and validation of an existing SP measure into native Spanish-speaking patients with schizophrenia. Given the good psychometric properties obtained by the Spanish adaptation, results further support the use of the SFRT-2 as an adequate measure to assess SP in patients with schizophrenia in both research and clinical practice. To that aim, SFRT-2 percentile scores for Spanish population were also provided in order to contribute to the appropriate detection of SP impairment in Spanish-speaking patients with schizophrenia.

### S61. THE ASSOCIATION OF VERBAL LEARNING DEFICITS WITH AGE AND SYMPTOMS IN SCHIZOPHRENIA

Dimitrios Kontis*[,1], Alexandra Giannakopoulou[1], Eirini Theochari[1], Angeliki Andreopoulou[1], Spyridoula Vassilouli[1], Dimitra Giannakopoulou[1], Eleni Siettou[1], Eleftheria Tsaltas[2]
*[1]Psychiatric Hospital of Attica; [2]Athens University Medical School*

**Background:** The relationship of age and symptoms with the performance on verbal learning and memory tasks in schizophrenia could provide useful information for optimizing and individualizing the efforts to remediate the cognitive impairments of patients.
**Methods:** During a cross-sectional study, 97 medicated and stabilized patients with chronic schizophrenia (61 males and 36 females, mean age=43.74 years, standard deviation-SD=11.59), which were consecutively referred to our Unit, were assessed using the Hopkins Verbal Learning Test (HVLT) and the Positive and Negative Syndrome Scale (PANSS). A linear regression analysis was conducted in order to investigate the effect of symptoms and age on HVLT performance.
**Results:** Increased age and total PANSS symptoms were associated with worse total recall (raw scores) (B=-0.109. 95% confidence interval-C.I.-=-0.18, -0.038, t=-3.038, df=90 p=0.003 and B=-0.053, 95%CI=-0.097, -0.008, t=-2.356, df=90, p=0.021, respectively). The effect of symptoms on HVLT total recall was significant for positive (B=-0.166, 95%CI=-0.316, -0.015, t=-2.189, df=90, p=0.031), negative (B=-0.167, 95%CI=-0.279, -0.054, t=-2.949, df=90, p=0.004), but not for general psychopathology symptoms (B=-0.05, 95%CI=-0.129, 0.03, t=-1.247, df=90, p=0.216). Further analyses revealed the significant negative correlations of total symptoms with the performance in immediate recall during the first HVLT trial (B=-0.021, 95% CI=-0.036, -0.005, df=89, p=0.011), and age during the second (B=-0.046, 95%CI=-0.076,-0.017, p=0.003) and third (B=-0.048, 95%CI=-0.083, -0.014, df=89, p=0.007) HVLT immediate recall trials. Both total symptoms and age were significantly negatively correlated with the performance in recognition discrimination (raw scores) (symptoms: B=-0.199, 95%CI=-0.363, -0.035, df=87, t=-2.415, p=0.017 and age: B=-0.357, 95%CI=-0.617, -0.098, df=87, t=-2.737, p=0.008). We failed to find any significant correlation between either age or symptoms with delayed recall.
**Discussion:** Age and symptoms are associated with immediate verbal learning and memory impairments but not with deficits in verbal delayed recall in schizophrenia. The effects of medication remain to be explored in future analyses. Cognitive remediation programmes against verbal learning deficits in individuals with schizophrenia should take into account their age as well as their symptomatology.

### S62. SUBMISSION WITHDRAWN

### S63. WHICH CLINICAL AND COGNITIVE FACTORS ARE RELATED WITH CHANGES IN JUMPING TO CONCLUSIONS IN FIRST-EPISODE PSYCHOSIS?

Susana Ochoa*[,1], Raquel Lopez-Carrilero[1], Eva Grasa[2], Maria Luisa Barrigón[3], ESther Pousa[4], Ana Barajas[5], Esther Lorente[6], Jordi Cid[7], Fermin González[8], Isabel Ruiz[9], Trinidad Pélaez[1]
*[1]Parc Sanitari Sant Joan de Deu; [2]Parc de Salut Marc; [3]Servicio de Psiquiatria y Psicologia Clinica; [4]Hospital del Mar; [5]Les Corts; [7]Hospital Clínic Valencia; [8]Institut de Psiquiatria de Girona, University of Jaen; [9]University of Málaga*

**Background:** The data gathering bias of jumping to conclusions (JTC) consists in a tendency to take a decision without sufficient information. There is evidence that suggests that the JTC bias does not improve (So et al., 2010), however other authors suggest that some psychological interventions such as Metacognitive Training have demonstrated that JTC can be reduced (Aghotor et al., 2010; Moritz et al., 2014; Pankowski et al., 2016; Ochoa et al., 2017). Nevertheless, any study has assessed the clinical and cognitive factor that are related with the improvement of this bias in schizophrenia or first episode psychosis.
The aim of the study is to assess which clinical and cognitive factors are related with the improvement of the JTC after a psychological intervention (Meta-Cognitive or psychoeducational group).
**Methods:** A total of 113 people were assessed with the beads task in two moments: basal and after 3 months. The sample was composed of people with a recent onset of psychosis, recruited from 9 public centers in Spain. Symptoms were assessed with the PANSS and the Psyrats; insight was assessed with the SUMD and the BCIS, and a neuropsychological battery including TMTA and TMTB, digits, WSCT and IQ was used.
**Results:** A total of 28 (24.8%) patients performed JTC in the basal assessment; of them 18 improved JTC after the interventions and 10 remains performing JTC. People who improved JTC presented higher levels of insight (p=0.032), better neuropsychological functioning in TMTA (p=0.011), Digits (p=0.033) and IQ (p=0.014). Moreover, people who improved JTC presented a tendency to score lower in hallucinations (p=0.097), and better in the WSCT (p=0.065) and TMTB (p=0.076).
